# Design and Development of a New Methodology Based on Expert Systems Applied to the Prevention of Indoor Radon Gas Exposition Risks

**DOI:** 10.3390/ijerph18010269

**Published:** 2020-12-31

**Authors:** Jorge Cerqueiro-Pequeño, Alberto Comesaña-Campos, Manuel Casal-Guisande, José-Benito Bouza-Rodríguez

**Affiliations:** Department of Design in Engineering, University of Vigo, 36208 Vigo, Spain; jcerquei@uvigo.es (J.C.-P.); jbouza@uvigo.es (J.-B.B.-R.)

**Keywords:** radon, expert systems, decision support systems, regression tree, risk, design science research

## Abstract

Exposure to high concentration levels of radon gas constitutes a major health hazard, being nowadays the second-leading cause of lung cancer after smoking. Facing this situation, the last years have seen a clear trend towards the search for methodologies that allow an efficient prevention of the potential risks derived from the presence of harmful radon gas concentration levels in buildings. With that, it is intended to establish preventive and corrective actions that might help to reduce the impact of radon exposure on people, especially in places where workers and external users must stay for long periods of time, as it may be the case of healthcare buildings. In this paper, a new methodology is developed and applied to the prevention of the risks derived from the exposure to radon gas in indoor spaces. Such methodology is grounded in the concurrent use of expert systems and regression trees that allows producing a diagram with recommendations associated to the exposure risk. The presented methodology has been implemented by means of a software application that supports the definition of the expert systems and the regression algorithm. Finally, after proving its applicability with a case study and discussing its contributions, it may be claimed that the benefits of the new methodology might lead on to an innovation in this field of study.

## 1. Introduction

### 1.1. Framework

Radon gas is radioactive, odourless, colourless and tasteless and belongs to the ‘noble gases’ group of chemical elements. It is produced by the radioactive decay of radium through an α-disintegration chain [1,2]. It emanates naturally from soil and rocks, penetrating into buildings thorough existing orifices (cracks, holes, etc.). It usually accumulates in enclosed spaces, presenting a half-life of 3.8 days for its most stable isotope [1,2]. The International System unit for radon gas concentration is the Becquerel-per-cubic-meter (Bq/m^3^) [3].

Several different studies consider that a radon gas concentration level between 5 and 15 Bq/m^3^, common in outdoor spaces, is not a health threat at all, and therefore its impact on health is usually considered only in enclosed spaces. In these places, the radon concentration values might often vary between 10 and 10,000 Bq/m^3^ [4] according to different environmental variables and conditioning factors, which might become a health issue [1]. Currently, inhalation of radon gas and its decay-products is one of the main causes of lung cancer [1,4,5,6,7,8,9,10].

In view of that, the harmful effect of exposure to radon gas on the health of individuals is proved. In particular, for the Galicia region in Spain—the geographic area where this methodology is being developed—several different works aimed to measure radon incidence in this zone are enumerated. In the current literature, a number of studies are available for that region, which is a high-radon incidence zone (more than 10% of dwellings where measurements were carried out by the Galician Radon Laboratory showed concentration values over 200 Bq/m^3^ [11]), aiming to connect those radon concentration levels to the incidence of several types of cancer. As an example, in the work by López-Abente et al. [12] an association was observed between the home concentration of radon gas and lung, stomach and brain cancer incidence in women. Along this same line, in the work by Barbosa-Lorenzo et al. [13] the existence of a correlation between lung cancer mortality in men and their home concentration of radon gas was concluded, once again making clear the impact of this gas on people’s health. In the PhD thesis of Castro Bernárdez [11] an analysis on the residential exposure to radon gas and lung cancer in the Ourense (Galicia, Spain) healthcare area was carried out. From it, it is concluded, in line with the previously introduced works, that an association exists between the concentration of residential radon gas and lung cancer incidence. Additionally, in the PhD thesis of Torres Durán [14] an analysis on the risk of developing lung cancer in a Galician non-smoking population exposed to high residential radon gas concentrations was performed. Its results indicated that people exposed to concentrations higher than 200 Bq/m^3^ show a risk of developing lung cancer that is 2.2 times higher than people exposed to concentration values lower than 100 Bq/m^3^. The aforementioned studies allude to the analysis of the effect of radon gas incidence on human health derived from its harmful concentrations in dwellings and residential and public buildings. Even if it is indubitable that there are industrial activities, such as underground mining, where radon concentrations are recognized as a considerable risk, the study and validation data used in this work came from the Galicia region (Spain), where there have been no underground mining activities for decades now. Because of that, at a social level preoccupation about the impact of radon gas on public health is focused on the study and adaptation of dwellings and residential and public buildings, on the light of a growing concern derived from the porous and granitic soils on top of which most of the Galician buildings are located [15,16].

Besides those already mentioned, there are different works that aimed to measure the impact of radon gas concentrations, both in the Galicia area and in contiguous zones such as the North of Portugal, which carry out studies oriented towards the processes for detection and risk prevention without highlighting the influence of radon gas on human health. In the work by Barros-Dios et al. [17] it was intended to determine which factors of a dwelling have an influence on its indoor radon concentrations. To carry out such a study, a measurement was made of the indoor radon concentration and the different characteristics were determined for a total number of 983 dwellings in Galicia. It was observed that the dwelling age, its building materials and the floor in which the detector was placed were related to the radon concentration levels measured. In the work by Quidós et al. [18], 300 gamma radiation measurements were made on Galician soils and 600 others on Galician dwellings, aiming to verify former studies that were carried out years before, and to establish a natural radiation map of the region. In the work by Cortina et al. [19] a series of home radon gas concentration measurements were carried out in the Santiago de Compostela area (Galicia). In this work, a correlation analysis was also performed between the radon concentration value and the location of the measurement stations, with no relevant relationship being observed. The authors of this work also collected measurements associated to meteorological parameters, obtaining a direct correlation between the indoor radon concentration value in dwellings and the derivative of the outdoor temperature with respect to time. In the work by Martins et al. [20], focused on the Amarante region (North of Portugal), a strong relationship was observed to exist between the soil characteristics and the measured radon gas concentration. In the presence of granitic soils, the observed concentrations are usually higher than those established as safe in the Portuguese regulations (400 Bq/m^3^). In this same line, in another work by Martins et al. [21] a study is made in the Vila Pouca de Aguiar region (North of Portugal as well). A geometric mean value of 568 Bq/m^3^ was observed for the radon concentration inside 91 dwellings, a value that was much higher than those from other granitic soil areas in the North of Portugal, a fact that highlights the need for making the population in the area aware of the risk that exists there.

Facing this impact, recent years have seen a clear trend towards an early and efficient prevention of harmful radon gas concentration levels, considered as harmful in residential buildings, in order to establish corrective actions that allow to mitigate its effects on the health of the exposed people [9,22]. In this sense, it is of interest to mention the work by Pereira et al. [23], focused on the design and implementation of a so-called ‘RnProbe’, an Internet of Things edge device for the integrated management of radon risk in buildings.

With the objective of providing an answer to the previously described problems, in this article a new methodology aimed to the prevention of the exposure to radon concentrations inside buildings that could result in harm to the health of the exposed people is presented and defined. Starting from the information provided by several sensors, the methodology allows providing recommendations related to possible corrective measures that might reduce the existing concentration of radon gas. To that end, a system is proposed that makes use of two expert systems working concurrently. Such systems are based in the use of fuzzy logic inference engines aimed to obtain outputs related to the gas exposure risk level and to the accuracy of that estimation. All these outputs, together with the sensors’ measured values history, are set as input data for a set of regression trees that, after being trained, allow to establish the degree according to which it is proposed the application of a collection of corrective measures—in a range of 0 through 5—associated to each one of them. This means to distinguish between recommendations for no action (0) and for compulsory actuations (5).

For the handling of this methodology the use of expert systems, considered as support tools for the corrective decisions aimed to the reduction of harmful concentrations of radon gas, was proposed. In essence, an expert system, defined within the scope of information systems and specified as a knowledge-based system, is a system that shows capabilities to find solutions to real projects taking into account a specific action domain, with a performance level that is similar to the one used by human experts [24]. Its scope is the improvement of the decision-making process by means of its capabilities for disseminating and formalizing information [25].

On this matter, the application of expert systems to problems related to the mitigation of harmful effects on human health or to improve environments that might be harmful to it—as it is the case of the ones used in this work—might be included within a wider approach related to the so-called ‘early warning systems’. However, this work does not address the definition of an early warning expert system, because it poses as necessary the human intervention in the interpretation of the decision derived from the methodology, as it is considered that the multiplicity of criteria intervening in the determination of the radon gas concentration is large and heterogeneous enough as to not deem useful a more autonomous approach in the decision making.

This work is organized and developed over five sections. In Section 1, the problems associated to radon gas are presented, together with the conceptual groundings on which the methodology is supported. In Section 2, a conceptual description of the design and definition of the methodology is made. After that, the methodology is proposed and described in detail. In Section 3, an illustrative case study is proposed that allows to understand the operation of the methodology. In Section 4, the discussion of the work is presented. In Section 5, the main conclusions obtained are presented.

### 1.2. Radon Related Concepts

Across this section several concepts are presented, related to the mechanisms that facilitate the flow of radon gas into buildings (Section 1.2.1), the criteria used for the application of corrective measures (Section 1.2.2) and the possible devices used to measure radon gas concentration (Section 1.2.3).

#### 1.2.1. Mechanisms and Factors That Boost Radon Leakage into Enclosed Spaces

Indoor concentration levels of radon gas may be influenced by multiple factors, among them atmospheric and seismic phenomena, architectural barriers, etc. [26,27,28]. Furthermore, perfusion of radon gas into buildings has been studied according to the basic mechanisms that favour its inflow into built spaces. There are basically two such mechanisms, the first one of them might be framed within the ‘gas diffusion’ mechanisms, and it is caused by the gradient of the radon concentration level that is present in the environment [10,28]. The second one, however, is caused by the gradient of atmospheric pressure that exists between the building envelope and the soil itself, and it is known as ‘advection mechanism’ [10,28].

In relation to the abovementioned factors, those that directly influence the increase of radon gas concentration inside buildings must be highlighted, and a classification can be made of them into two groups: internal and external factors [10,29].

Internal factors refer to those features that are inherent to the building itself and its placement, such as the construction materials, the ventilation system installed, or the soil on top of which it is placed, among others [10].

Among the external factors there are meteorological phenomena (rainfall, relative humidity, atmospheric pressure, temperature, and wind direction and speed) [10,28,29,30,31,32], seismic movements and atmospheric instability [10,29].

As regards to the factors associated with rainfall, it has been verified that high amounts of it on a dry ground could result initially in a reduction of the radon concentration level on the upper soil layers because of the gas solubility [29]. If the heavy rainfall persists, the soil could reach concentration levels close to saturation, establishing a boundary that might avoid radon gas leakage towards the surface [29]. Additionally, and taking into account the yearly seasons, it has been observed that radon concentration is usually higher in winter than in summertime [29,33]. In the cold nights and seasons (Autumn-Winter), the temperature difference between the internal and external home spaces is at its maximum [33]. That is why an atmospheric pressure gradient appears between the indoor and outdoor spaces of the building, causing the radon to leak in and to accumulate inside it because of the stack effect [10,28,33,34]. In this sense, different studies associate the use of hearths and fireplaces to an increase in radon gas concentration because of its depressurization effect and the increase of the air-exchange ratio, but without obtaining a clear relationship [35,36,37]. When time slots are taken into account, it is observed that radon concentration in homes is higher during the night than in daytime [33], showing maximum values early in the morning and minimum ones in the evening [10,29]. Such differences are caused by a smaller activity level of the residents during the night, and therefore to the existence of smaller ventilation rates [33]. Furthermore, besides the studies referenced in Section 1.1, it might be worth to highlight that the factors associated to the people’s uses and habits can play a fundamental role in the increase of the radon gas concentration. Thus, the indoor ventilation rate, the use of air conditioning devices, or the behaviour of the residents at home might result in noticeable differences between regions. In this matter, the cultural and social differences are also key factors and they must be taken into consideration when studying radon gas issues [38,39].

As a general rule, the existence of mechanisms and multiple facts that might change the radon concentration level inside a home may be concluded [26,27]. Both the diffusion and the advection mechanisms may be corrected by means of preventive actions. Internal factors are usually constant, with little sensitivity to changes with time, unless any construction modification is made on the building. External factors, however, show a higher variability across the year, are more sensitive to changes, and therefore their influence in radon concentration changes is higher.

In any case, the reduction of the radon gas concentration must be a national priority to be addressed in the building codes and the regulations affecting the residential use of buildings, because of its indubitable influence on the health of its inhabitants. To have available a methodology that is easy to apply and reliable in its recommendations could entail a better processing of the determination of the risk derived from exposure to the gas and in the adoption of measures addressed to a better handling of enclosed spaces, and to the improvement of the dwelling habits related with ventilation and air treatment. All that will contribute to a better quality of life for the inhabitants, not only reducing their exposure to radon gas and therefore the risk of suffering its associated diseases, but also mitigating its long- and medium-term effects and serving in turn as an awareness-raising measure. In one word, the presented methodology would provide an efficient mechanism for solving the associated problem, while at the same time it reduces and monitors the risk of radon gas on public health by means of its prevention and processing, and thus it would tune in with the Agenda 2030 as a conveying tool for its goals [40].

#### 1.2.2. Criteria for the Application of Corrective Measures

Among the increasing body or rules and regulations related to radon gas control, the EU’s, USA’s and World Health Organization’s (WHO) recommendations may be highlighted. According to the United States Environmental Protection Agency (EPA), corrective measures should be applied to reduce concentration levels above 4 pCi/L, a value equivalent to 148 Bq/m^3^ [22]. Despite that, it also indicates that values below 148 Bq/m^3^ might involve some heath risk, this being in line with the WHO proposals on the topic, which establishes a reference level of 100 Bq/m^3^ [1] in order to minimize the potential impact on the health of people exposed to indoor radon gas concentrations. In those cases where it is not feasible to set that limit because of—among other factors—ambient conditions, WHO indicates that the concentration level should not exceed 300 Bq/m^3^ in the worst-case scenario. According to EU Directive 2013/59/EURATOM, the reference level for radon concentration in indoor work environments should not exceed a mean concentration level of 300 Bq/m^3^ on a yearly basis [41]. That regulation is of compulsory application by all EU member states since February 2018. Many countries have developed their own particular safety rules and criteria for the prevention and treatment of long-term exposure to radon gas, which in general terms agree with the WHO’s recommendations [42].

#### 1.2.3. Radon Gas Detection Systems

Even though in this work a system for the detection of radon gas is not specifically developed, the presented methodology incorporates readings from these systems in its operation. Because of that, it is deemed convenient to contextualize the most well-known detection systems. There are nowadays several different technological approaches that provide an answer to the need of a process for determining radon gas concentration levels. From a functional point of view, radon gas detectors might be distinguished attending to their need for an external power supply in their operation. According to this criterion, a first general classification is made into active and passive detectors, the first of which need of an external power supply for their operation while the second ones do not need it. This first general classification [1] is complemented by a second one that establishes a distinction between active sampling and passive sampling detectors; for those in the first group the air sample is forcefully collected by means of a pump, while in the second group the air sample is naturally collected, by means of diffusion of permeation effects [43]. It is worth to mention that both classifications are complementary, being possible for example to find active detectors that allow active or passive sampling. In the same way, and in order to avoid confusion among those two classifications, a third one might be considered between detectors capable—or not—of real-time detection, related as well with the measurement method used [44]. Together with these general considerations, there are still other classification criteria, that may be distinguish between ‘short-term’ when their needed time of exposure to radon gas is reduced—from a few seconds to a few hours—and ‘long-term’ when their exposure to the gas needs of several days, or even months. This time-based classification may be elaborated in more detail based on the already pointed-at measurement method, by which three main groups can be established. Thus, it is possible for example to find detectors that use grab sampling methods, which perform instantaneous measurements in short periods of time, from 1 to 20 min. Another group uses continuous sampling methods by automating the measurements in short reiterated intervals across long exposure periods of time. Lastly, the integrative sampling methods collect data associated to the radiation effects—such as air ionization—across periods of time ranging from a few days to a few months, calculating then the mean values [44,45]. Thus, according to the aforementioned classifications and taking into account their most identifying characteristics, in Table 1 a summary is made of the main technologies used for radon gas concentration measurement.

### 1.3. Use of Expert Systems in Decision-Support Tools

Generally, any methodology that arises from the interpretation of information aiming to provide support to the decision-making process is susceptible to be considered within the information systems overall framework. This kind of methodologies, shaped as decision support systems and offering huge versatility and adaptability capabilities [49,50,51,52] have come to constitute in these last decades its own discipline, framed as mentioned before within the scope of information systems. Decision support systems have the capability for being used in multidisciplinary environments, facilitating the use and integration of different techniques and methods to allow an efficient management of information. Among these are the gradual integration of the advances derived from data science and artificial intelligence in the shaping of expert systems, which not only allow to drive the decision-making process, but also to solve problems in real environments, incorporating the knowledge from different sources and providing the capabilities that are needed to improve the decision-making process [25].

These expert systems started to be developed in the 1960s [24,53,54] and have capabilities for translating and transferring human knowledge and experience into simulation and calculation environments [54,55,56], thus providing answers to complex problems. Their use, therefore, always within the frameworks of information systems and knowledge-based systems, is articulated as complementary or component tools in the usual methodologies that provide support to the decision-making process [57,58,59,60,61,62]. For their correct and complete definition it is necessary to start from an expert knowledge base, a human-machine interface, an inference system and a set of data from the problem represented on a real dominion [24,53,55,56,63,64,65].

On the other hand, from the point of view of design within information systems, it is essential to consider design science as established in the works by Hevner et al. [52,66]. Design science may be applied to the building of software artifacts that allow providing solutions for information management, whatever their purpose will be. These artifacts will implement the structures of the decision support methodologies and, in the same way, will accommodate the expert systems themselves. Therefore, this is in essence what is proposed in this work, the design and development of a new decision support methodology applied to the prevention of the exposure to harmful concentrations of radon gas, articulated by means of a software artifact that will implement the set of expert systems and decision algorithms.

In a way that is similar to the traditional engineering applications, the design within information systems encompasses a process that must start from the identification of a problem, as well as the establishment of needs, until finally obtaining a product, in this case being the previously mentioned software artifact that allows meeting the requirements and limitations of the problem. In this work, and previously to the generation of the artifact, the guidelines proposed by Hevner et al. [66] are analysed, which allow one to evaluate the design of the artifact itself.

#### 1.3.1. Expert Systems Applied to the Interpretation of External Factors and Environmental Conditions

The implementation of the formerly described methods may come together with the use of an expert system in the role of manager of the collected data. It is possible to find in the current literature a large number of works related to the interpretation of environmental conditions and phenomena. The work by Brambley et al. [67] in the 90s presents and describes an expert system, the Expert Radon Mitigation Advisor (ERMA) developed by the Pacific Northwest Laboratory. The user inputs information about the conditions of the place to be monitored in the system, such as for example the type of heating installed, as well as an historian of measurements of radon concentration level at the place. From that information, the system provides the user with recommendations about the most appropriated method to mitigate the radon gas risk. In a more general way, the work by Reffat et al. [68] proposes an expert system aimed to the evaluation of the environmental quality of a specific room, taking into account multiple factors such as thermal comfort or air quality, among others. The work by Shoom and Bowen [69] discusses the design of the Indoor Air Quality testing and evaluation Expert System (IAQES), an expert system that aims to diagnose problems existing in indoor spaces related to air quality, and to suggest potential solutions. On another line, for example, the work by Ikram and Qamar [70] poses and implements an expert system oriented to earthquake prediction. In the work by Shin-ya et al. [71] it is intended to improve the air quality inside a building by means of the use of expert systems. The authors developed a tool oriented towards the identification of 9 polluting elements—CO_2_, CO, NO_2_ or radon, among others—inside a building. This tool, besides diagnosing the presence of the contaminants, also provides support to the process for designing the ventilation systems. Fang et al. [72] use GEOTOX, a tool developed in their previous works [73,74,75,76], as an expert system that aims to help to evaluate hazardous waste, allowing to perform interpretation tasks, i.e., to assess places that present a potential hazard to health. In the thesis of Byrne [77], a hybrid knowledge-based advisory system is developed to be applied to the mitigation of radon gas risk. Such system takes into account, from the selection of the corrective means—for example, fans—up to the determination of building materials and the estimation of costs.

As a collateral—but not necessarily conjoint—use of expert systems, the so-called ‘early warning systems’ are worth mention. Widely used and applied in fields as heterogeneous as health [78], natural disasters [79] or finance [80], this type of systems aims to provide a quick and anticipative answer to any fact considered as harmful. Their traditional definition might characterize them as information systems that encompass means for data (sensor) reading and processes for detection, decision-making, information transmission and answer-generation, aiming to minimize the impact of a harmful or pernicious fact [81]. From this point of view, the presented methodology might be understood as an early warning system; however, in this case the main goal is not only to minimize the damaging effect from harmful radon gas concentrations, but also to establish its causes and to propose a reasonable solution to the detected problem. That is why an expert system is proposed, i.e., a system having the capability for finding solutions to real problems within a domain of specific action and with a performance level similar to human experts of technicians. It is not proposed, therefore, a simple detection based on the identification of precursor effects, but a methodology is defined and developed that aims to describe the problem and, with its application, to go looking for an optimal solution to it, adapting itself to the criteria, viewpoints and specific scenarios of its use. Once this expert system has been specified, and its information dissemination and formalization capabilities have been verified, then it would be possible indeed to integrate it into an early warning system, thus defining a so-called ‘early warning expert system’ [82].

### 1.4. Decision Trees

Decision trees define a set of predictive methods used in the fields of statistics, data science and machine learning. They are grounded in the determination of different groups, by means of which the space of initial data–predictors might be segmented of fragmented, in order to look for models in them that allow to link them by means of a common class—or prediction variable. They allow, therefore, to omit global models with multiple predictors, reducing them to local models by looking for a characteristic in the data that will give a determination of groups as pure as possible, that is, groups where all the predictors will be associated to a single class. The final objective will be to find a distribution that returns groups as pure as possible.

The algorithms that implement decision trees use non-parametric approaches and fit within the category of supervised learning techniques. Depending on the nature of the prediction variable, they are called regression trees or classification trees. A common feature to all of them is the use of some metric that allows to measure the purity of the groups, i.e., the homogeneity between the data correspondence and the class they are assigned to. Using a general notation, it is about measuring the purity of each set by means of a metric, that might be linked to the concept of entropy of a random variable [83], that determines the degree of purity for the different groups and allows to establish a criterion to stop the splitting. It will be the metrics for this purity measurement, understood also as criteria for performing subsequent splits, together with other rules for stopping the formation of new groups, which will distinguish among the different algorithms.

There are nowadays several algorithms that are used to support the predictive methods based on decision trees. Iterative Dichotomiser 3 (ID3), extended by C4.5, the multivariate adaptive regression splines (MARS), Chi-square automatic interaction detection (CHAID), and the classification and regression tree (CART), are among the most widely used and proved algorithms [84,85,86,87,88].

In this work, the use of CART-type algorithms is proposed for obtaining a regression model. Initially developed in the 1980s by Breiman et al. [84], the CART algorithm defines a set of binary decision trees where the space of predictors is getting subsequently split into two groups, aiming to find the most pure ones: those that are associated to a single class. In the last years, the use of CART-like algorithms has become widespread because of their simple software implementation and the better knowledge about them by researchers [89]. CART presents advantages with respect to other regression and classification algorithms, such as its non-parametric nature (it doesn’t take into account the distribution of the values of the predicting variables), as well as its capabilities for finding splitting variables and for managing both outlier data and missing variables. Furthermore, the implementation of CART has been greatly automated and its interpretation is relatively simple, even without having a specific training on decision trees [89].

Therefore, the CART decision tree consists in a binary and recursive partition procedure [32]. As it may be observed in the work by Lemon et al. [90], which shows a basic schematic for a decision tree, this gets defined by a series of nodes and branches. Starting with the ‘root’ node, the group containing all the predictors, and after examining the different input data or independent variables, a first split is established with two descending branches that give place to two new nodes—two groups that divide the initial space—named as ‘child’ nodes. In a similar way, the different independent variables continue to be examined and new splits are made from the previously obtained child nodes, producing new branches [91]. Finally, the terminal nodes—or ‘leaves’—are determined, which establish sub-groups from the initial sample that are mutually exclusive [90]. As it has been already mentioned, the splitting process will stop when the nodes reach a purity level that minimizes their entropy, meaning that they contain data corresponding to a single class.

Depending on the nature of the dependent—or predictor—class or variable, classification or regression approaches may be chosen [85]. For the former, the Y domain within which the predict variable y moves is a set of non-ordered values, i.e., a category such as: ‘winter, ‘night’ or ‘rainy’, while for the latter the predict variable y moves within a continuous domain Y that takes real values, i.e., ‘date’ (expressed as day of the year), ‘time’ (expressed over 24 h) or ‘rainfall’ (expressed in litres of water by square meter) [84,85,92,93]. Resuming the description in the last paragraph and considering this difference, the CART algorithm applies different metrics, such as successive splitting criteria, when it is used either as a regression or as a classification model. As a regression model, it usually applies the ‘impurity’ concept, which relates to the Residual Sum of Squares (RSS) that in essence looks for a distribution of the predictors–independent variables—space in different classes—or regions in this approach—aiming to lower the sum of its impurities as much as possible. The RSS metric, in turn, is defined as the sum of the squared deviations between the dependent variables values and the mean value for the region. The generic expression for impurity if defined in Equation (1) [94], where $y_{k}$ represents the value of the dependent variable and $\bar{y}$ is the mean value for the region [84,94]. On the other hand, when classification models are concerned then the metrics applied are usually the Gini or Towing [85], where Gini is defined as a measurement of the variance in the set of the different node classes:

(1) $$\mathrm{Impurity}\;\mathrm{of}\;\mathrm{the}\;\mathrm{nodes}\;=\overset{m}{\underset{k=1}{\Sigma }}{\left(y_{k}\;-\;\bar{y}\right)}^{2}$$

In this specific case a choice is made for a regression tree. The sequence that is necessary to determine it might be defined according to the next stages [84,93,94]:

- Step 1: Start from the root node that contains all the independent variables.
- Step 2: For each independent variable x ϵ X, find all the potential splitting points by defining a series of regions or nodes that do not overlap each other, and that are associated to the observable values linked to the predictor variable in each one of those regions.
- Step 3: For each one of the previously identified nodes, the subset *S* that allows one to minimize the impurity of the node into two descending branches giving place to two child nodes, must be found. As it has already been mentioned, the impurity parameter represents the sum of the squared deviations between the dependent variables’ values and the mean value for the region or node [85]. A group might be considered as pure when all its elements belong to the same region, and as impure when all of its elements belong to different regions [85]. When a node is pure, it might be said that a terminal node—or leaf—has been reached. However, when a node is impure it is necessary to determine if it is wished to stop and accept the obtained group, or else continue performing splits considering other independent variables [85]. As it has been already mentioned, there are several indices to measure impurity—such as Gini for classification problems [85]—but in this case, because a regression case will be applied, the RSS metric becomes important, aiming to its minimization as a metric for more homogeneous nodes.
- Step 4: The former process is repeated recursively through the recursive binary splitting method, selecting the predictor variable and the splitting point that guarantees a lower total RSS value by using the subsequent nodes until the tree reaches the maximum size that was assigned to it.
- Step 5: If a stop criterion is reached then the iteration is halted; else the command will return again to Step 2. In relation to the stop criteria, it is necessary to be careful because an early stop might result in a tree that is very small as to represent the structure of the starting data. On the contrary, a late stop might produce a tree that is too large, or even unstable, and with no useful meaning whatsoever [85].
- Step 6: Finally, after obtaining the value associated to a prediction line, it is proceeded to calculate the root-mean-square error (RMSE) in order to determine the accuracy and precision of the proposed regression model, by calculating the square root of the mean value of the sum of the squared differences between the predicted and the actual values. That is, starting from the initial data, a measure is established of how far away the predicted and the actual values are [95].

There are other possible approaches, based on the idea that using stop rules might cause important associations in data to get lost because of an early stop, and thus they propose to build an extremely large tree that accommodates all the possible levels, to be later pruned using several strategies [90,91].

## 2. Materials and Methods

### 2.1. Definition of the Methodology

#### 2.1.1. Previous Considerations

As it has been already mentioned, and prior to the presentation and conceptual description of the methodology, it is necessary to validate its development by using, in this case, the proposal made in Hevner et al. [52,66], based on a set of criteria that must be met by the contributions within the field of information systems.

In Table 2 a review of the degree of achievement of the proposed methodology according to the guidelines proposed by Hevner et al. is presented [52,66].

#### 2.1.2. Conceptual Design and Description of the Proposed Methodology

In this work a new decision-support methodology is designed and defined, based in the use of two concurrent expert system combined with regression trees. All that is applied to the prevention of exposure to harmful radon gas concentrations inside buildings, especially aimed to those having a high occupancy, such as healthcare buildings. As it has already been commented, the design of the proposed methodology is formulated as the design of a software artifact that meets the requirements and limitations of the problem to be addressed. In this sense, the design process must start with the collection of needs, summarized in Table 3, from which it will be possible to establish the technical specifications and the design constraints.

**Figure 1:**
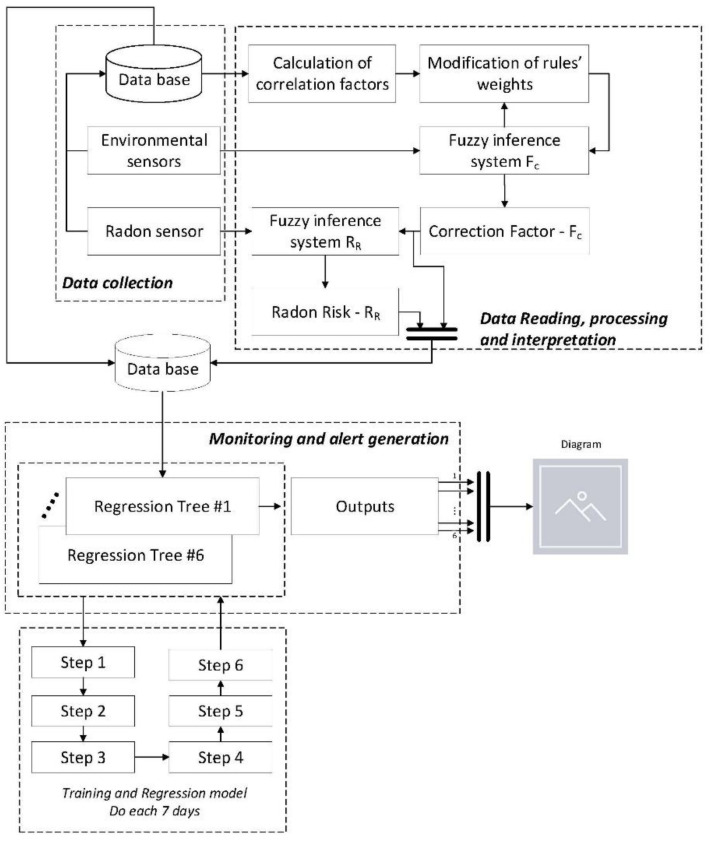
Flowchart of the initial methodology.

Figure 1 shows the basic flowchart of the decision-making support methodology, which will be described next, encompassing the whole of the information handling from the data collection to the recommendation of corrective actions after the evaluation made by the different inference systems.

The methodology is based on the use of two concurrent expert systems [96,97,98] for the calculation of the radon risk value, together with a decision tree-based regression model, that will determine the final recommendations associated to such risk. All of them are respectively labelled in Figure 1 as ‘Fuzzy inference system Fc’, ‘Fuzzy inference system RR’ and ‘Regression Tree #Number of Tree’. Each one of these expert systems is provided with a Mamdani-type inference system [99,100,101,102]. Mamdani’s inference method is one of the most popular ones in literature [102]. It is characterized by its graphical representation of an inference process, that is, given some previous facts or hypothesis (antecedents), it is possible to obtain or infer a derived fact (consequent) starting from linguistic rules based on syllogisms. Using these natural language rules of the IF-THEN type, the Mamdani fuzzy inference system allows to combine the different antecedents of each rule and to determine its consequents, these to be later aggregated to obtain a real-number output value by means of a defuzzification process. The representation of antecedents and consequent is carried out by means of the well-known membership functions—the mathematical representation of a fuzzy set—that determine the degree of membership of a certain value to a particular set. The subsequent combination and aggregation of antecedents and consequents uses different specific operators that ensure the graphical combination of the membership functions [96,98,102].

The first of the Mamdani inference systems in the methodology feeds, on the one hand on the data collected by a set of environmental sensors, and on the other hand on the data loaded from a database. The second inference system has as input the reading of the radon system and the correction factor F_c_ obtained from the first inference system. The combination of both will generate the radon risk index R_R_. Each one of the decision trees is fed the previously established factors—F_c_ and R_R_—and the different data collected, as well as the history of recommendations associated to all those data. From them, each one of the mentioned trees allows one to establish the degree of recommendation associated to a certain corrective and preventive measure which allows, either to reduce the radon concentration, or to produce an alert on the inconsistency in the prediction.

##### Data Collection

The methodology starts with the periodic collection of information by means of different data provided by sensors. All these data, together with those derived from the methodology itself, will constitute the knowledge base. Such data will be compiled through a control unit to be later processed through a filtering and classification system, analogous to the one described in the article by Blanco-Novoa et al. [3] or the one proposed by Font and Baixeras [26]. The implementation of the sensor system, as well as the collection, gathering and transmission of data, is a previous step to the development and application of the methodology presented in this article. Even so, and in a general way, it is possible to consider that the data will come from two different sensor families:

- Environmental and atmospheric sensors.
- Radon sensor.

##### Data Reading, Processing and Interpretation

Once the input data for the system is available, their manipulation starts within the methodological process itself. Also, the data collection may be performed concurrently with their processing. The methodology is developed in a way that does not depend on the type of data collection system used, and it is possible to adapt and apply it to any sensor set and to any information pre- and post-processing hardware system, that might be either commercial solutions or specific developments.

To start with, the data—both those coming from environmental and atmospheric sensors—is collected to be later stored into a database. After a relevant number of data collection cycles, it is possible to establish the correlation coefficient that exists between the radon concentration level and the different environmental and atmospheric variables, from the values stored into the database. After that, processing is carried out on the data collected by the two Mamdani-type fuzzy-logic expert systems that work in a concurrent way [96,97,98]. The first system is provided with a feedback from the historian as Figure 1 shows, in which depending on the correlation existing between the radon concentration level and the value of the different atmospheric variables, the values of the different rules of the system itself are weighted in one or another way. The outcome of this first system is a factor that aims to express what will be the future trend of the radon concentration level, known as ‘correction factor’ (F_c_). In the second system, the calculation is performed and an index value related to the risk implied by the radon existing in the building is obtained, known as ‘Radon Risk’ (R_R_).

##### Monitoring and Alert Generation

Subsequently, by means of the interpretation of the radon risk and as a helping tool to preventive decision, recommendations will be generated relative to the application of corrective measures that could allow to minimize the health risks derived from extended exposure to those concentration levels. Such recommendation levels would be the outcome of a regression model based on the CART algorithm, the correction factor value, and the environmental and atmospheric data, and determines a set of outputs that are to be understood as recommendations to be followed in order to minimize the health risk associated to the radon concentration level. These outputs would be grouped in the one of the following two categories:

- Preventive actions
  ○ Activate forced evacuation and mechanical ventilation.
  ○ De-activate forced evacuation.
  ○ Natural ventilation.
- Checking actions
  ○ Check sensors.
  ○ Check inference.
  ○ Check exposure time.

**Figure 2:**
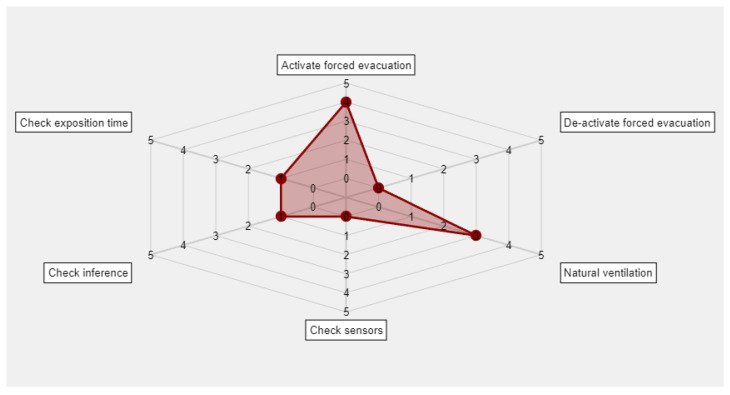
Example of a recommendation diagram.

Therefore, from the decision trees a total of six outputs are obtained that are represented in a graphical way in order to provide support to the decision to be made. The chart aims, precisely, to highlight the non-unquestionable nature of the posed recommendations and to stress their need for interpretation that, in this case, falls on the user of the methodology. It is they who must assess its convenience and degree of application, as well as its conjoint nature, establishing the appropriate corrections on the final measures that, if they exist, must be collected on the application database. In this sense, for example, the proposed outputs are not unchangeable, but they must be studied and validated before the implementation of the methodology is made on a study zone. If the final user considers necessary to modify any of them, then the code of the software artifact that supports the execution and monitoring of the methodology must be consequently modified as well. Figure 2 shows one of these representations, where in a scale from 0 to 5 the strength of the recommendation to be followed is specified. Such scale is standardized within this interval in a way that these recommendations can be compared appropriately. Both the preventive actions and the checking actions are established within the quantitative range from 0 through 5 units. They must not be interpreted as complementary to each other, but the value that each one of them takes within the aforementioned numerical interval aims to make evident the degree in which those actions must be applied. That is, absolute states are not intended, in which for example a full forced evacuation activity might be given, but intermediate states are intended in which such evacuation is carried out in a more or less exhaustive way. We proceed in the same way with the rest of the actions. This capability for graduation connects with the need for interpreting the actions to be followed, because the methodology performs recommendations that must be necessarily interpreted by the final user.

### 2.2. Implementation of the Methodology

The methodology that was introduced and developed in the previous section encompasses several stages that allow performing the collection and processing of data by means of expert systems that act as complementary decision-support tools. Next, the operation of such methodology is explained in detail.

After the sensor data—both the atmospheric- and the radon-related—are collected, the information produced is stored and structured into a database to be processed later. This information will constitute the knowledge base for all the expert systems and, together with their outputs, will make up the training dataset for the regression trees. Starting then from the building of the knowledge base, an initial processing is carried out that consists in the calculation of a number of coefficients that aim to represent the correlation that exists between the environmental variables and the radon concentration value, that is, to determine the influence of the rate of change of the atmospheric parameters on the change of the radon concentration over time. These coefficients are in turn reinterpreted to be applied as modifiers for the importance level of the different rules used by the first expert system in the methodology, which is based on a Mamdani-type inference system [99,100,101,102]. In this way, it is achieved to adapt such system to the specific circumstances for each location where the radon risk value is in need to be assessed. In the same way, it is also achieved to narrow the uncertainty level that is associated to the methodology and to expert systems, both in their interaction, random and epistemic variants [103].

Later, the assessment of the information coming from the atmospheric factor in the previously mentioned first information system is carried out, obtaining as its result a factor, named as ‘correction factor’ (F_C_), that aims to express how favourable or unfavourable are the atmospheric conditions in relation to producing a rise or fall in the radon gas concentration value. Concurrent to this first expert system, a second system operates that is in charge of the assessment of the risk that the current radon concentration value inside the building may involve. Taking into account both the effect of the atmospheric conditions through the correction factor and the technical measurement of the radon concentration, it is obtained as an output of the inference process the parameter named as ‘radon risk’ (R_R_). This risk value aims to be an objective metric of the danger that a permanent presence into the monitored zone has on the health of people. It is a proper metric, because of the defuzzification procedure that assigns to it a value that is tangible, measurable and comparable in future measurements. It is objective as well, because it not just takes into account the measurement of the radon sensor, but corrects it depending on the environmental effects which their non-linear relationship with the radon gas concentration has been modelled by using the first expert system.

Finally, by means of a decision tree-based regression model that the atmospheric variables, the current radon concentration value, as well as the F_C_ and R_R_, are fed into, it is possible to calculate the recommendation level associated to each one of the corrective measures listed and suggested in the methodology. Next, the operation of each one of the components in the methodology will be explained in detail.

#### 2.2.1. First Inference System—Correction Factor

As it has been mentioned before, the correction factor (F_C_) is the output of the first expert system, which is a Mamdani-type fuzzy logic-based inference system [99,100,101,102]. Such system, shown in Figure 1, is in charge of assessing the data coming from the environmental sensors.

##### Calculation

**Figure 3:**
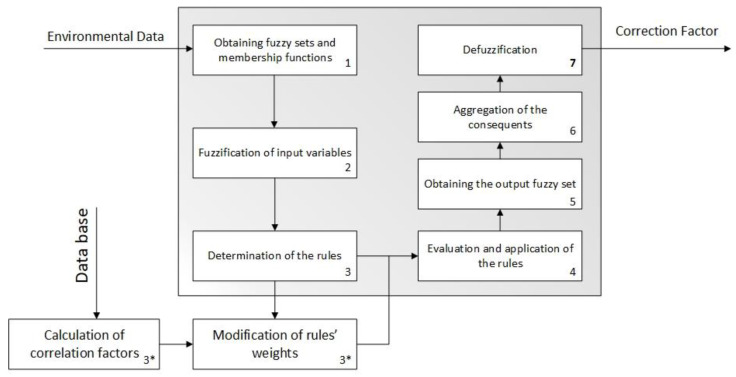
Inference system used for the Correction Factor. The steps where the correlation factor is calculated in order to modify the rules of the inference system are indicated with a *.

The correction factor (F_c_) is obtained as an output of the defuzzification process performed by the first inference system. Figure 3 shows the detailed schematic of this Mamdani-type inference system [99,100,101,102]. The inference process starts with the fuzzification of both the input and the output quantitative variables by means of their corresponding membership functions, previously defined but always subject to modification from the software artefact configuration options. Trapezoidal functions [102] are used both for the input and for the output variables, because it is considered that there will always be a range of values showing maximum membership to each one of the qualitative valuation scales in which the different functions are divided into. In the case of the input variables, the valuations will be grading qualifiers (low, medium, high) within an interval determined by the maximum and minimum values of their corresponding technical measurement ranges. In the case of the output variable, i.e., the correction factor, its qualifiers will coincide with those of its antecedents and the value interval will be [0, 10].

Once this is done, the different fuzzy values obtained are combined according to a set of pre-established combination rules. The elaboration of the rules will not be conditioned by subjective values and will be limited to two antecedents for each rule because of the easiness to propose and compare the effects [104,105]. Their combination is carried out by means of AND-type operators [102], as the consequent needs of the combined participation and contribution of all the antecedents. The output of this process is an aggregation of the output functions associated to each rule that is finally defuzzified, thus obtaining a specific value that in this case is the correction factor. Prior to the aggregation of the consequents, that will follow a disjunctive approach [102], the implication must be carried out of the consequents which, when an AND operator is applied to the antecedents list, will be determined by truncation using the minimum value of those obtained in the membership functions of the antecedents in the application of the rule. After the aggregation of the consequents, the final value of the correction factor value is calculated by means of the defuzzification process based on the centroid method [102]. In the work by Casal-Guisande et al. [96] a more detailed explanation of the development of these systems is provided.

As a particular feature of this inference system, it must be pointed out that in the initialization of the rules a standard weight of 0.25 is assigned to each one of them. Additionally, such weight may be altered by means of the calculation of a correlation coefficient established between the atmospheric conditions and the radon concentration. As the data is collected by the sensors, such data gets registered on a knowledge base, thus allowing to create a history from which it is possible to calculate the existing correlation, as already mentioned, between the atmospheric variables and the radon concentration. The different rules in the system are weighted depending on the correlation coefficient obtained, giving more importance to those in which the variable that they depend on shows a high correlation level to radon concentration. As the correlation coefficient may either have a positive or a negative value, it is important that all the possible rules have been defined previously, even those that might result a priori to be either evident or contradictory. For example: IF (Temperature-Difference is High) THEN (Correction-Factor is High) and IF (Temperature-Difference is High) THEN (Correction-Factor is Low). Thus, it will be the system itself the one that, by modifying the weight of the different rules, determines which ones of them will have a higher or lower weight.

##### Determination of the Correlation Coefficient

For the calculation of the correlation coefficient between two variables (atmospheric variable and radon concentration), in this case it is proposed the use of the Pearson correlation coefficient. This coefficient measures the degree of lineal dependence that exists between two variables, taking values in the range [−1, 1] [106,107]. When its value is zero, there is no relationship whatsoever between the considered variables. Conversely, the close its value is to one, the higher the relationship between the considered variables will be.

The knowledge base will be updated after each one of the sensor data readings, and the Pearson correlation coefficient between each one of the different atmospheric variables and the radon concentration will be then re-calculated. Equation (2) shows the expression of the coefficient for two variables, *a* and *b*, where E(a,b) is the co-variance and $\sigma_{a}$ and $\sigma_{b}$ are respectively the variances for those variables [107]:

(2) $$\rho \left(a,b\right)=\;\frac{E\left(a,b\right)}{\sigma_{a}\cdot \sigma_{b}}$$

The values obtained for the correlation coefficient belong to the closed interval [−1, 1]. This indicates a linear behaviour of the relationship between the atmospheric factors and the radon gas concentration, which not always corresponds to real cases, in which there could be non-linear growth episodes related to both variables for a specific time period. Aiming to model this behaviour, a transformation is applied to the correlation values obtained, that allows estimating non-linear behaviours in them, as well as fast rises and falls within measurement intervals. Such transformation is based in the use of a sigmoidal function that, starting from the data within the interval [−1, 1] (values to be shown in the abscissa axis considering their absolute value), determine values corresponding to the interval [0, 1] from the ordinate axis by means of the application of a sigmoidal function that is fully defined by the user of the methodology. Thus, in summary, starting from the Pearson correlation coefficient values and considering their absolute values, these are defined within the interval [0, 1]—it must be pointed out that those values close to 1 or −1 determine clear proportionality relationships—of the abscissa axis of a sigmoidal function. The ordinate axis for such function is defined within the interval [0,1] and represents the weight or graduation of the combination rules of the first inference system where the variables considered in the calculation of the different Pearson correlation coefficients take part. Because of that, depending on the correlation coefficient value obtained and after applying the sigmoidal transformation, the weights for the system’s rules will be modified. This entails that, for example, if the value obtained in the transformation is higher than zero, then the estimated correlation between the radon concentration and the atmospheric variable is—either directly or indirectly—proportional, meaning that the rules in which these variables are related will be prioritized according to that proportion. In the same way, a value of zero will be assigned to those rules for which the variables are related by a proportionality that is different to the one established by the correlation coefficient. The graduation in the correlation coefficients is represented by the sigmoidal transformation, allowing modelling both the growth rate and the position of the inflexion point on the abscissa axis, thus reflecting the correlation between the variables by modifying their initial values. In this way, it is possible to modify the non-linear behaviour of the correlation coefficients with regard to its assignation as weightings for the combination rules from the inference system that will determine the final correction factor. Equation (3) shows the generic expression for a sigmoidal function, where the parameter *a* allows to modify its amplitude in the ordinate axis, *b* represents the growth rate of the sigmoid function, and the parameter *c* allows to move the sigmoid middle zone with respect to the coordinates origin along the abscissa axis. As the value of the weight presents a maximum value of 1, the value of *a* will be 1. On the other hand, the values for the parameters *b* and *c* may be established by the user themselves in the configuration stage. Thus, for example, the modification of the parameter *b* allows modelling the relationship between the environmental factors and the radon gas concentration by changing the growth rate, with which the user may opt for a smoother of steeper transition between the low and high weighting zones. The modification of the parameter *c* allows, for example, to keep the sigmoid inflection to the extreme values in order to only weight combination rules having membership values that are equally extreme. In this way, the act of moving the sigmoid towards the left will cause the system to be more sensitive to changes, while if it is moved towards the right the opposite effect would be obtained. Equation (4) shows the expression used for the calculation of the new weight:

(3) $$sigmoid\left(x;a,b,c\right)=\;a\cdot \frac{1}{1+e^{-b\cdot \left(x-c\right)}}$$

(4) $$new\text{\_}weight\left(\rho ;b,c\right)=\frac{1}{1+e^{-b\cdot \left(\rho -c\right)}}$$

In the definition of the rules, different levels of a consequent for a certain antecedent are initially considered. For example: Rule 1—IF (Temperature-Difference is High) AND (Radon-Concentration is High) THEN (Correction-Factor is High), and Rule 2—IF (Temperature-Difference is Low) AND (Radon-Concentration is High) THEN (Correction-Factor is High). If the correlation coefficient between the indoor-outdoor temperature difference and the radon concentration is positive, then the value of the new weight of Rule 1 will be the one determined by Equation (4), while the new weight of Rule 2 will be zero. This means that a direct proportionality between the ‘Temperature-Difference’ and the ‘Radon-Concentration’ variables is considered to exist, which a priori might appear to be true, but not conclusive. Because of that, the use of the sigmoidal transformation makes sense, as it allows correcting the correlation coefficient according to the expert’s criteria. In this way, the system will be able to adapt itself and to determine which ones are the atmospheric conditions that show a higher influence on the radon concentration.

With all that has been previously explained, the user has full control on the final determination of the correlation coefficients, which in turn represent a weighting of the combination rules for the first inference system that is in charge of the determination of the correction factor. This control will not always be mandatory as it will depend on the assessment that the user sees fit, according to their experience and the information cumulated into the methodology’s database, with respect to the interpretation of the initially calculated correlation values.

#### 2.2.2. Second Inference System—Radon Risk

**Figure 4:**
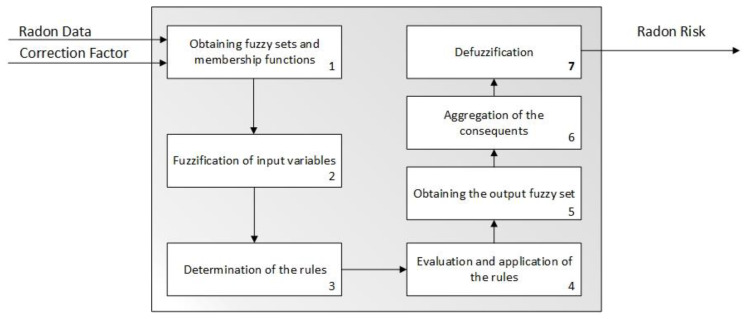
Inference System for the Radon Risk.

The radon risk (R_R_) is the output of the second expert system which, as was the first one, is a Mamdani-type fuzzy logic-based inference system [99,100,101,102]. Such system is in charge of quantifying the existing risk from the combination of the radon concentration reading and the correction factor obtained from the first inference system. Figure 4 shows a schematic in which the different stages for the calculation of the radon risk are established. It is an inference system that starts with the fuzzification of the input variables, or antecedents, and output variables, or consequents, by means of the previously defined membership functions. Same as in the case of the first inference system, the antecedents use a trapezoidal-type function with a technical interval for the case of the radon gas concentration and an interval [0, 10] for the correction factor. In this case the consequent is the radon risk value, represented by a trapezoidal function defined in the abscissa interval [0, 100] with a reasoning that is similar to the one followed in the definition of the correction factor in the first inference system. After being combined according to a set of pre-established rules, it is proceeded to the aggregation of the output functions associated to each rule, or consequents, and eventually to the defuzzification of the final aggregated set. As happened in the first inference system, the combination rules are defined without external conditions and are combined using AND-type operators. The implication of consequents, therefore, will truncate its own membership function by the minimum value of the membership functions of the antecedents, proceeding to it aggregation by means of a disjunctive method that fuses the envelopes of the consequents of all the rules.

The output of this system, after applying the centroid method to the aggregated function of the consequents, is named as radon risk (R_R_). This risk reflects a measurement of the harmful effects on people’s health of an extended exposure to radon gas, measurement that is associated both to the atmospheric conditions and to the reference location the methodology is applied to. The determination of this risk by means of an inference system allows reducing the uncertainty associated both to its definition and to its determination. The presence of relevant radon gas concentrations must not imply in all cases that a risk associated to the exposed person exists, but that the impact of those concentrations must be interpreted as a probability of causing harm. In the present methodology, this expression of probability is represented as a risk percentage, derived as explained from quantitative values and therefore, in principle, not in need of interpretation or of a qualitative factor to maximize or minimize these values. It is, accordingly, an estimation of the danger of being exposed to radon gas concentrations that do not address directly the discretionality in the exposure time, as all those qualitative considerations are integrated into the definition of the factor.

#### 2.2.3. Regression Trees and Generation of the Spider-Web Diagram—Establishment of Recommendations

So far the correction factor and the radon risk have been calculated, obtained after the defuzzification of their corresponding inference systems, which work concurrently [96,97,98]. Such information must be structured into a knowledge base, together with the values of the atmospheric variables, the radon concentration, and the level of each one of the corrective measures associated to each dataset (variables and concentration values). Overall, in this methodology it is posed the use of 12 independent variables and a dependent variable, associated to each one of the six recommendations, for each one of the regression trees used. The independent variables are the following: Year date, Day hour, Radon concentration, Indoor temperature, Outdoor temperature, Atmospheric pressure, Wind speed, Relative humidity, Correction factor and Radon risk. As mentioned before, the dependent variable associated to each decision tree is a quantitative measurement associated to each one of the six actions that are determined as outputs of the methodology. With that, it is intended that the relationships between the atmospheric variables, the radon concentration and the radon risk with respect to the recommendations followed in order to reduce harmful radon gas concentrations, would be modelled. The recommendations suggested by this methodology correspond to different actions that must be carried out, either in person or remotely, in the zone to be monitored. As it has been already mentioned in the *Monitoring and alert generation point*, the recommendations made cover the scope of corrective and preventive measures, as well as the potential detection of errors in the measurements. The set of quantitative measurements is established as a prediction set (dependent variable) of the decision tree, providing a real value laying into the interval [0, 5] for each one of the determined actions. On the other hand, all the initial information mentioned before is quantified in a direct or indirect way and implemented as either explaining or independent variables from the tree itself. Two inferred variables are also added, these being the correction factor and the radon risk, that also encompass an expert interpretation of the environmental factors and radon concentration values mentioned before. With all that, it is intended that the assessment of the action is as rigorous as possible, fitting its value to trusted concentration estimations and radon risk values. With the progressive use of the methodology, the data collected and corroborated will improve the training of the trees, and therefore also improve the proposed regression model.

The use of decision trees as recommendation predictors is not only related to the proved capability of these algorithms for finding a relationship between heterogeneous data without any apparent link, but also because of their easiness of interpretation and their low need for pre-processing the initial data. Even if the regression models show a certain loss of information in the categorization process, and there is a trend towards unbalanced trainings in the presence of predominant classes, in this work its use presents differential advantages. The initial data derived from the measurements and the subsequent outputs from the inference systems will be fed into the CART algorithm without a specific pre-treatment, while the classes will be obtained from the recommendations for actuation facing the data collected. As it has been already mentioned, the recommendations are grouped into two large sets: ‘Preventive Actions’ and ‘Checking Actions’, each one of them containing three sub-sets. These six sub-sets, that will act as prediction variables, will be evaluated within an interval of [0, 5] according to the degree of need for their implementation. In this way, an initial data package will have 6 outputs that will correspond to a continuous value within the closed interval [0, 5]. Each one of these outputs will determine a single regression tree, being therefore necessary to train and cross-check six trees associated to a single output that matches each sub-set.

From all this information, it is possible to train each one of the six regression trees based on the CART algorithm. The initial data are common for all of them, while the different output classes are structured as continuous values in the interval [0, 5] associated to each recommendation sub-set. The measures will be collected into the knowledge base with a frequency of twice per day, proceeding to train the trees each week, that is, starting with the first 14 data lines collected the algorithm will be trained when 14 new registers are available. Once the tree is trained, it is possible to deduct the recommendations in each one of the two samples that are collected daily. To do that, the trained tree will be gone over according to the new initial data—the new predictors—and the estimated output value will be determined, composed of a set of six natural numbers that are associated to each one of the six recommendations listed and proposed in the methodology. In order to verify the accuracy of the proposed regression model, monitoring and following up the root-mean-square error (RMSE) will be performed as a measurement derived from the mean difference between the values predicted by the regression model and the actual values taken from the starting data [95].

Finally, the recommendation provided by the tree will be expressed as a spider-web diagram, thus making possible to determine, in a graphical and intuitive way, which are the specific actions that are recommended for the user to perform. These recommendations require an interpretation, individual and combined, from the user of the methodology, who is in charge of their implementation from a larger viewpoint that includes multiple criteria, points of view and application scenarios.

## 3. Case Study and Results

In this section, the application of the methodology on a case study is shown. In order to facilitate its monitoring and understanding, a software artifact was implemented using the MATLAB^®^ R2020b platform [108]. The inference systems have been implemented using the Fuzzy Logic Toolbox [109]. Additionally, an integral graphical interface was developed using the App Designer module. As regards to the generation of the spider-web diagram, a third-party script was used that is available at Github [110].

### 3.1. Data Collection

**Figure 5:**
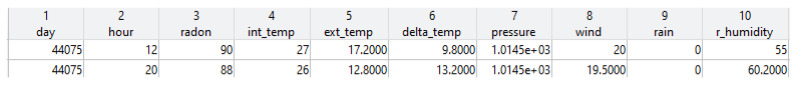
Data header.

As it was mentioned previously, the methodology starts with the collection and the storing of information into a database carried out by different sensors. The elaboration of this case study starts from a series of data packages collected twice a day, at 12.00 and 24.00 h, for a full month. In this case study, a choice was made for carrying out two control measurements per day, which in any case might be modified by the user if they see it fit. Figure 5 shows how the collected data are structured: date of measurement, time of measurement, radon concentration in Bq/m^3^, indoor room temperature in °C, outdoor temperature in °C, indoor-outdoor temperature difference, atmospheric pressure in mbar, wind speed in km/h, rainfall in mm, and relative humidity in %. As regards to the data in the ‘day’ column, a pre-processing was made on them so that the value ‘01 September 2020’ is coded as the integer ‘44075’, the value 02 September 2020 as ‘44076’, and so forth.

### 3.2. Data Reading, Processing and Interpretation

**Figure 6:**
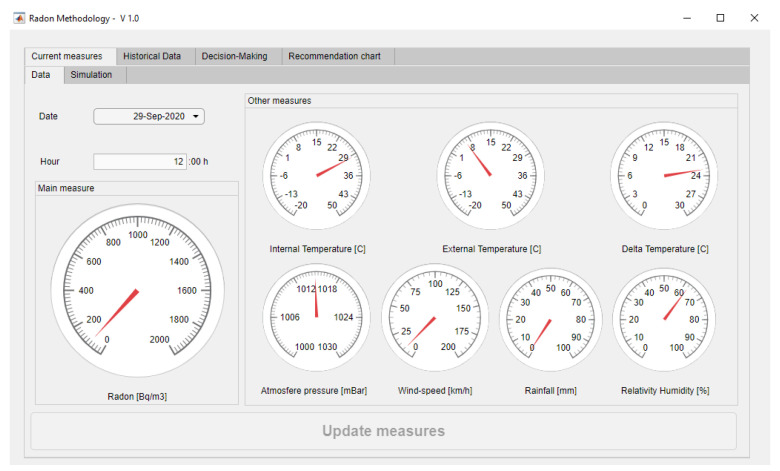
Dashboard for the visualization of the variables.

As the sensor measurements are collected and structured into a database, it is possible to carry out a reading on them by the system. Figure 6 shows a dashboard that allows displaying the last measurement data. It consists of two clearly differentiated regions: a first block in which the main measurement—the radon concentration value—is shown, and a second panel which displays the measurements of the atmospheric sensors. Additionally, the date and time of measurement are shown.

In this specific case, as it can be deduced from Figure 6, the values from the last measurements collected from the sensors on date 29 September 2020 at 12.00 h are as follows:

- Radon concentration value: 84 Bq/m^3^.
- Indoor temperature: 29.6 °C.
- Outdoor temperature: 6.5 °C.
- Atmospheric pressure: 1014.8 mbar.
- Wind speed: 9.5 km/h.
- Collected rainfall: 1.2 mm.
- Relative humidity: 62.3%.

The previous data will be used to predict the recommendations suggested by the methodology, once the regression trees are trained.

**Figure 7:**
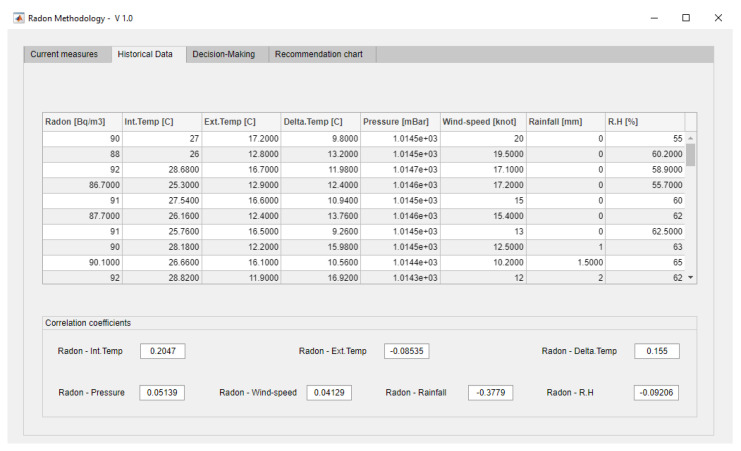
Dashboard for the calculation of the correlation coefficients.

From the data history it is possible to calculate the correlation coefficients that exist between the radon and the atmospheric conditioning variables. Figure 7 shows the dashboard for the software in charge of the calculation of the correlation coefficients from the history data. In this case, the correlation coefficients obtained between the different values are the following:

- Correlation coefficient of radon concentration vs. indoor temperature: 0.2047.
- Correlation coefficient of radon concentration vs. outdoor temperature: −0.08535.
- Correlation coefficient of radon concentration vs. temperature difference: 0.155.
- Correlation coefficient of radon concentration vs. atmospheric pressure: 0.05139.
- Correlation coefficient of radon concentration vs. wind speed: 0.04129.
- Correlation coefficient of radon concentration vs. rainfall: −0.3779.
- Correlation coefficient of radon concentration vs. relative humidity: −0.09206.

It is observed that one of the strongest correlations is the one that exists between radon concentration and rainfall, in this case having a negative sign, which may be interpreted as that when the rainfall value grows the radon concentration value drops, and vice versa.

**Figure 8:**
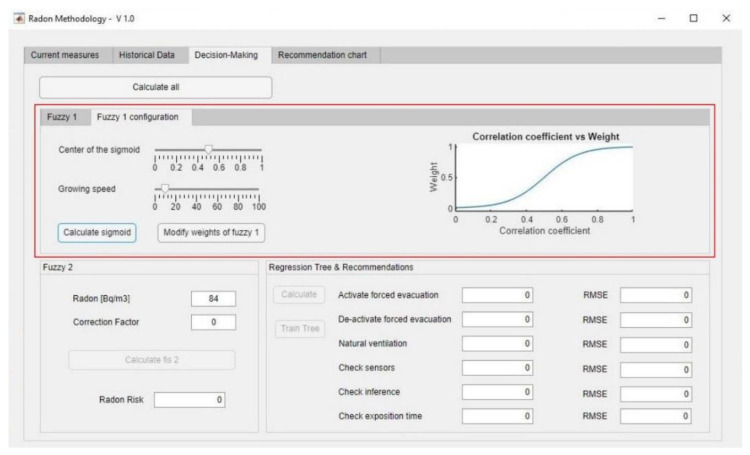
Dashboard for the definition of the sigmoidal function.

These correlation coefficients, after they have been transformed by means of a sigmoidal function, are used to modify the weight of the rules from the first inference system. Figure 8 shows the dashboard used for the modification of the sigmoidal function parameters that allows to decentre it and to change the growth rate, and therefore the weights to be obtained. This will have a great influence on the correction factor to be obtained, making the system to be more or less sensitive to the changes associated to the variables that are the most dominant on this phenomenon.

**Figure 9:**
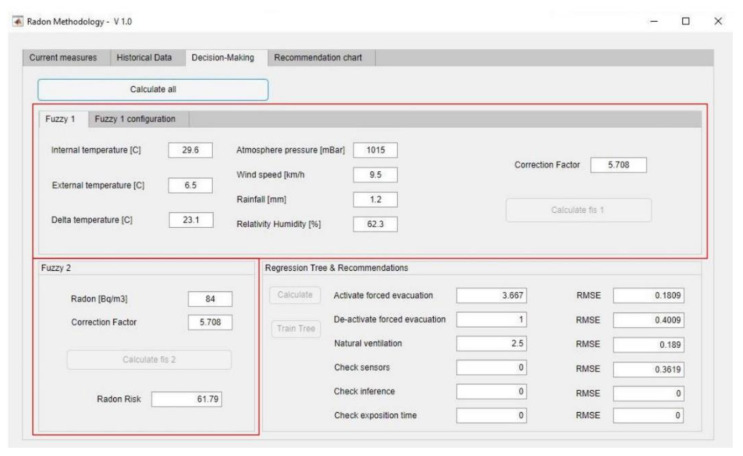
Dashboard for inference systems 1 and 2.

Once the correlation coefficients have been calculated and the rules of the first inference systems have been modified, it is proceeded to the calculation of the correction factor and the radon risk values by the inference systems 1 and 2, respectively. In Figure 9 the calculation blocks of the inference systems 1 and 2 are highlighted on the graphical interface. A correction factor value of 5.078 and a radon risk value of 61.79 were thus obtained. If the sigmoidal function was displaced towards the left, then the correction factor value would be higher.

### 3.3. Monitoring and Alert Generation

The data collected after the defuzzification process performed in both inference systems, together with those previously collected, are structured into a database. The first time the system is used, in the stage just before its autonomous operation, the user must set the levels associated to each one of the recommendations, thus establishing the training dataset for the regression trees. After that, when the system is in steady-state operation, the trees will be trained again each time 14 new lines are incorporated into the database, i.e., each seven days. In this case, with the data from the last registered measurement shown in Section 3.2 of this case study, the following recommendation levels have been obtained:

- Activate forced evacuation: 3.667/5.
- De-activate forced evacuation: 1/5.
- Natural ventilation: 2.5/5.
- Check sensors: 0/5.
- Check inference: 0/5.
- Check exposure time: 0/5.

**Figure 10:**
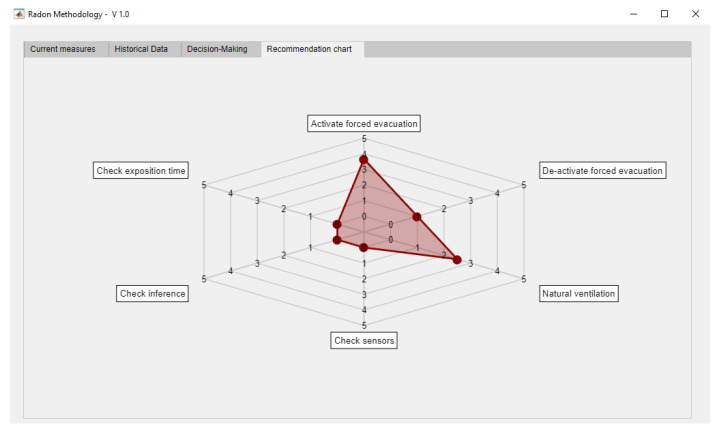
Spider-web diagram.

The RMSE for the sample displays values of 0.1809, 0.4009, 0.1890, 0.3619, 0 and 0 that correspond to each one of the six proposed regression trees. As it happens that all of these values are close to zero, and taking into account the measurement scale, the regression values are considered overall as reliable because they show a small difference between the actual and the predicted values [95]. As a consequence of that, and aiming to ease the interpretation of data by the user, these are displayed in the form of a spider-web diagram, as shown in Figure 10.

In the view of the recommendations obtained, it may be concluded that the current radon gas concentration is prone to rising, and because of that it is recommended to activate the forced ventilation as a first option, or else to choose natural ventilation solutions.

## 4. Discussion

In the present paper a novel decision-support methodology related to the prevention of the harm caused by exposure to radon gas is proposed. Such a methodology is grounded on the integration of expert systems into the process for the determination of the risk associated to the radon gas concentration level, combined with a regression model that will determine the degree in which the implementation of some corrective recommendations is necessary. The issues associated to radon gas are unquestionable, as already described in Section 1 where, additionally, the main detection technologies are identified. The use of expert systems allows increasing the validity of these technologies, as they allow combining the data obtained through them with those derived from different atmospheric sensors, in a way that by means of inference engines the interpretation of the results obtained by the radon gas concentration measurement devices might be improved. In this sense, the proposed methodology aims to optimize the components that constitute the measurement system in a way that does not depend on the arrangement and/or the type of elements used for the collection and processing of information. That is, the arrangement of the sensors and other hardware elements is not a decisive factor in the application and development of the methodology, which always will make use of the data stored into its knowledge base to carry out the risk inference calculations and the final establishment of recommendations. However, this independence does not downplay the importance of a correct design of the hardware elements in charge of providing such data. Thus, as already mentioned in Section 2.1.2, starting from a trusted arrangement, composed for example of a set of active radon detectors together with a weather station, experimentally validated [26], the methodology could add a further recommendation to the measurements obtained, not only optimizing their interpretation but also detecting potential trends, biases or errors associated to the implementation of the measurement system itself. The contribution does not lie in the choice for a certain sensor, but in the implicit capability of the methodology for inferring a potential risk associated to high gas concentration values by means of the expert systems and their capabilities for the estimation of recommendations from a set of data. According to the improvement of the inference capabilities of the expert systems, the fine-tuning of the correlation coefficient and the data stored, the methodology will steadily achieve more reliable predictive functionalities even if the measurement system might become unreliable and indicate gas concentration values far from the actual ones. Thus, the initial methodology may keep optimizing the rules of the inference engines by means of the data stored, in such a way that the control of the results is progressively getting improved. Finally, the methodology provides a diagram of recommendations that is determined from all the data processed: atmospheric sensors, radon detector, and risk and correction values, derived from the use of regression trees based on the CART algorithm. This algorithm allows to find a non-linear relationship between all the input data and to establish a level of need in the interval [0, 5], associated to six different corrective recommendations. These recommendations are grouped into two sets: on the one hand the preventive ones that aim to lower harmful gas concentrations, while the checking actions, on the other hand, aim to alert of an incorrect or failed operation of the methodology and its supporting hardware. This last aspect must be highlighted, as the methodology is robust before the possibility of detecting errors in the measurement sensors. Besides the monitoring that the user might perform on the different values collected or inferred, there is an inherent control in the application of the CART algorithm that allows detecting potential errors. In the development of the first inference system, the calculation of the correlation coefficients would provide a first warning about a potential malfunction in the radon sensor to the user of the methodology, because the Pearson coefficient values would differ much from those recorded in the history data base. In the case the user does not notice it, the system would carry the error and would produce an erroneous correction factor value that would in turn result in a radon risk value that is also in error. Thus, when those data were inserted as a prediction line for the CART algorithm, depending on the stored history two approaches show up, the first one of them associated to very early stages of the application of the methodology, and therefore with a low number of records, where the regression model obtained would provide results with a very low reliability, represented by a high root-mean-square error (RMSE) value. The second one, associated to an advanced stage of application, would have a high number of records and in it the regression model would univocally determine a check sensors action, as it would connect the prediction data with other similar data from the past that determined at that moment, after the review of the results, the existence of errors in the radon sensor. This involves reinforcing the idea on the non-dependence of the methodology with respect to the system of sensors and other measurement hardware. As an example, many studies highlight the relationship that exists between indoor radon gas concentration and indoor-soil pressure difference [28,111] which might lead to the recommendation of incorporating a specific sensor. Even if this metric were not initially considered, the evolution of the results, drifting away from the actual conditions, would cause errors in the accuracy of the CART algorithm derived from a wrong determination of the correlation coefficients. That would force the users of the methodology to check, not only the status of the sensors but also their appropriateness to the radon risk modeling by analyzing and interpreting the collected and inferred sets of results. Thus, the incorporation of a specific sensor aimed to collect a particular value would be straightforward for the user, as he would need only to adapt the membership function that corresponds to the first inference system, and to update accordingly the table of correlation coefficients.

The combination of the inference systems with a regression tree is one of the differential aspects of the presented methodology. The main features and contributions of each one of these approaches have been already pointed to in the paragraph above, so now a combined analysis of them will be addressed, considering the methodology within its field of study: the analysis, prevention and reduction of harmful concentrations of radon gas. As described in Section 1.3.1, there are different approaches that relate the use of expert systems with prevention systems. When adding to these the ones categorized as early warning systems (described in the aforementioned section), then a big enough corpus of knowledge would be available on which it would be possible to carry out a comparison. Thus, the points of the methodology that might be highlighted when considering their combined operation would be the following:

- Unlike early warning systems, the use of expert systems combined with decision trees is not aimed only at minimizing the impact of radon gas concentrations, but it also intends to mitigate the existing effects and to enrich the knowledge base of the system, in a way that allows to keep inserting and identifying factors influencing those gas concentrations.
- When defined as expert systems, the decisions will always be subject to a final review because, even if they may be understood as multi-criteria decision methods, in no case they encompass all the criteria, viewpoints and scenarios that could show up. So, its preventive effects would be a consequence of its evolution as knowledge-based systems, while their derived decisions will always be interpreted by the users, who at any moment may modify them, either inside or outside the methodology.
- The incorporation of modification mechanisms that are not related to the re-programming of the software artifact is one of the key points of the methodology. It clearly increases its versatility of use as well as its adaptation to different scenarios and circumstances. Said scenarios, in principle related to buildings with a residential use and their associated environmental metrics, may be extended with a mere re-definition of the membership functions and the dependent variables, defining through these all the specific circumstances for each scenario where the methodology is wished to be applied. For example, if its use were focused on underground mines then it would be necessary to re-define the intervals and qualifiers of the input variables to the first inference system, as well as removing and considering recommended actions according to their origins. In any case, those changes would be easily implemented.
- The necessary interpretation of the results derived from the algorithm establishes the nature of expert systems, enriches their knowledge base, and incorporates a qualitative control on the uncertainty that is associated to the definition and application of the methodology. With all that, the uncertainty that is inherent both to data collection and to their qualitative interpretation is reduced. Not only the uncertainty from the randomness of measurements is reduced, but also the epistemic is—this one related to the lack of information—and the one related with the own interaction of the systems, algorithms and data collection processes as well. The fact of supervising the risk determination by means of a correction factor, and the constant monitoring that is provided by the definition of the membership functions, allows an expert user to continuously improve the prediction capability of the methodology.

The results derived from the application of the methodology suggest, not only that the needs established in Table 3 are met, but that they have been improved. The presented interface, even if it is liable to further modifications to improve it, is clear and easy to use. The collection of information, besides the calculations related to the inference system and to the CART algorithm, are autonomous and do not require any user intervention. However, even if the functional aspects of the software artifact are obvious, the results obtained also invite one to consider an unquestionable reliability in its prediction capabilities. The final suggestion resolves that it is necessary a corrective measure such as the—forced or natural—ventilation, as the methodology predicts a trend towards harmful concentrations of radon gas. The fact of having established a relationship between rainfall and radon gas concentration allows making predictions based on atmospheric variables, controlled by means of regression trees. For example, in the case study, when both a trend is observed of rainfall reduction and a medium value of radon gas concentration is detected, combined with an increase in temperature difference, the methodology recommends preventive ventilation.

The use of expert systems as a support tool for decision-making is common in engineering processes and, of course, a highly evolutionary and wide field of study. There are nowadays applications that make use of the expert system concept in many fields, from the civil to the health environment. In this sense, the use of expert systems allows to diversify the collected information in such a way that it may be interpreted in a more rational and normalized way. It also contributes with a feature to formalize the information to optimize the risk value inferred by the methodology. It is precisely in this aspect where the presented methodology distinguishes itself from other ones applied to environmental systems. It is known the capability of expert systems to provide reasonable solutions to undetermined problems. The already mentioned capabilities for diversification and formalization of information are a key difference in the decision support methodologies based on these systems. By complementing these features with the use of regression trees for establishing an assessment of the recommendations to be followed before a certain radon gas risk value, a methodology has been built that is robust, efficient and differential. The authors are not aware of any instance of the combined use of expert systems and regression algorithms in the field of decision making related to the identification of harmful radon gas concentrations. There are some works, already commented in Section 1.3.1, that suggest the use of expert systems aimed to that same purpose, but the authors themselves point to the future development of proposals that have been already included in the presented methodology. As an example, Fang et al. [72] in their 1993 work pointed to the use of correlation models as complementary to the predictions, besides highlighting the need for using several expert systems to improve the acquisition of all the process information. In the same sense Brambley et al. [67] take a stand by stating that the use of expert systems applied to establish processes for radon gas mitigation need of an efficient data collection at several different levels of application. All the previously mentioned works, as well as those referenced in Section 1.3.1. are useful as a development base for the methodology proposed in this article and make it possible to identify and contrast the presented improvements.

## 5. Conclusions

Both the radon gas detection technologies and the devices that implement them constitute nowadays an industry with its own entity because of the undeniable health problems associated to the exposure to high concentration levels of this gas. By proposing a new methodology that will provide support to the decisions related to the prevention of high concentrations of radon gas, a system for the prevention of the risk associated to the exposure to radon gas with an increased effectiveness and reliability has been defined. The use of expert systems combined with decision tree algorithms means a clear differentiation of the presented methodology with respect to other ones that exist in the field of study. Because of that, the proposed methodology not only presents a determined and measurable control on the different types of uncertainty that exist in information systems, but also allows establishing an objective measurement of the radon gas risk. Finding a reliable metric for the risk of this gas, associated also to a graduation of recommendations to be followed, makes possible to size the real use of the proposal made. Precisely, that usability is shown in the case study, where it is highlighted the capability for stating the relationship between different variables, first with radon gas concentration and second with the proposed recommendations. It may be highlighted that all those features can always be adapted to each specific user, either directly or indirectly, by means of the definition of the software artifact. Considering all these aspects, the methodology presented in this work makes possible a continuous improvement in the process for preventing exposure to harmful radon gas concentrations, focused especially on dwellings and public and residential buildings. Its implementation is not only recommended from the viewpoints of engineering and healthcare, but it connects directly with the sustainable development goals included in the Agenda 2030, specifically in reference to ‘ensure healthy lives and promote well-being for all at all ages’ [40], as it allows an efficient reduction in a potential risk to public health, as well as a future integration—as justified before—into an early warning system.

Even so, there are still several limitations that must be subject to an important future development. Expert systems show a strong dependency on both their knowledge base and the inference engine used. This issue, together with the difficulties that the decision tree-based regression model shows when the class variability is low, makes compelling to improve the processes for the definition and the conceptualization of both the inference models and the regression algorithms. In the same way, the use of both rule systems with two antecedents, even if it eases its interpretation, at the same time limits the identification of existing correlations between environmental variables. New approaches where an efficient dynamic effect exists in the elaboration of the membership functions and the definition of combination rules with several antecedents, together with the use of forest models or support vectors, might improve the capability for prediction of the current methodology.

## Figures and Tables

**Table 1:** Main technologies currently in use for the measurement of radon concentration values.

Technology	Air Sampling	Measurement Technique	Duration of Studies	Description of the Technology
Activated carbon detector	Passive	Integrating [44]	Short-term studies (periods generally shorter than one week)	These are based on the capability of active carbon to retain radon gas [46]. After their use, the amount of gamma radiation emitted may be determined in a laboratory.
Electret ion chamber detector	Active and passive variants	Integrating	Depending on the ion chamber design, it may allow to carry out short-term or long-term studies [47]	This type of devices measure the ionization produced when radon atoms disintegrate inside the chamber [47].
Track-etch detector	Passive	Integrating [44]	Long-term studies	After exposed, the detector is chemically and/or electrochemically etched and analysed in a laboratory to obtain the average radon concentration from the density of tracks produced by radon and its progeny [46].
Scintillation cells	Active	Grab or Continuous [44]	Commonly used for carrying out continuous measurements [3] for both short and long cycles	It consists of a cylindrical device coated with a luminescent material [47], with one of the walls being transparent, and activated by alpha emissions [46]. The emitted photons are captured and amplified by a photomultiplier tube [46,47], thus allowing to determine the radon gas concentration level in air [46].
Gas-filled detector	Active and passive variants	Grab or continuous [48]	May be used both for short and for long measurement periods	It is a device in which an interaction is produced between the radioactive particles derived from the radon gas present in a chamber, generating ion couples that are attracted to charged electrodes [47]. It may use different gas mixes such as air, argon with a small amount of methane, and argon or helium with small amounts of any halogen element [47].
Solid-state detector	Active and passive variants	Grab or continuous	Can be used both for short and long measurement cycles.	It is based on the interaction of the emitted radiation with a semi-conductor material that produces electron-hole couples that are then collected by charged electrodes [47]. Depending on the type of design it may focus on one or another radiation type, with germanium and silicon being the most commonly used materials [47].

**Table 2:** Verification of the guidelines in Hevner et al. [52,66].

Guideline 1: Design an Artifact (the Proposed Methodology)
The artifact, meaning the methodology as detailed in Section 2.2, consists in a helping tool aimed to the process for the prevention of high indoor concentrations of radon gas. In the first place, the calculation is made for the correction factor, from the information collected by a set of sensors, by means of a fuzzy logic-based inference system. Later, said correction factor is combined with the current radon gas concentration within a second inference system, also based on fuzzy logic, from which the radon risk level is obtained. Finally, from the calculated values and the data history it is possible to train regression models based on a decision tree, that will be used to perform recommendations on the levels at which the corrective measures should be applied to reduce the concentration of radon gas. With the objective of automating the calculations and facilitating the understanding of the proposed methodology, the implementation of the system has been carried out into a software artifact defined using the MATLAB^®^ environment developed by The MathWorks, Inc, Natick, MA, US.
Guideline 2: Relevance of the problem
The problem derived from the inhalation of radon gas results nowadays to be unquestionable, because it is recognized as the second-leading cause of lung cancer after smoking [1,5,6,7,8,9,11]. That is why it becomes very important the development of a methodology that allows to detect and anticipate potential situations in which the current radon gas concentration might get increased inside a building.
Guideline 3: Assessment of the design
The application of the new methodology is shown in the case study described in Section 3.
Guideline 4: Contributions to the field of research
The contributions to the field of expert systems are presented in Section 4 and Section 5 of this article.
Guideline 5: Rigour in the research
The conceptual development of the presented methodology, together with its classification within the field of investigation, has been defined in Section 1. In the same way, the mathematical foundations of this work are supported on the use of fuzzy inference systems, given their proved effectiveness and their capability for handling uncertainty in decision-making processes.
Guideline 6: Design as a search
In Section 1, the methodology has been framed within the state of the art that is inherent to the field of study.
Guideline 7: Communication of the research
In Section 5, the main contributions of the new method are presented, as will be the future lines of work.

**Table 3:** Design needs, requirements, and their corresponding restrictions.

Prevention of Exposure to Harmful Radon Gas Concentrations		
Design Needs for the Artifact	Technical Requirements	Restrictions Associated to the Environment
The methodological process, from data collection to its interpretation, will need as little user interaction as possible	Programming the sensor data reading, and the automatic filtering and labelling of information Programming the autonomous-operation inference models	Errors or corrections that need the intervention of an expert
It must have an interface where the recommendations are shown in a graphical way	Graphical interface	The user must have available a device to run the software
It must collect environmental information and store it in a convenient way	Definition of a knowledge base supported by common database systems	The applicable restrictions from the devices and the environments where the software is implemented
It must process recursively the information	Continuous reading/writing on the database systems	None
It must calculate a risk value associated to the exposure to radon gas	Conjoint implementation of the inference systems together with the regression tree	Limitations associated to the algorithms themselves

## Data Availability

All relevant data is available in the present manuscript. Technical specifications and code are available on request from the corresponding authors.
